# Efficient Generation of Chondrocytes From Bone Marrow–Derived Mesenchymal Stem Cells in a 3D Culture System: Protocol for a Practical Model for Assessing Anti-Inflammatory Therapies

**DOI:** 10.2196/42964

**Published:** 2023-07-28

**Authors:** Rajashree Patnaik, Shirin Jannati, Bala Mohan Sivani, Manfredi Rizzo, Nerissa Naidoo, Yajnavalka Banerjee

**Affiliations:** 1 Mohammed Bin Rashid University of Medicine and Health Sciences Dubai United Arab Emirates; 2 Department of Molecular Biology Lund University Lund Lund Sweden

**Keywords:** chondrocytes, bone marrow–derived mesenchymal stem cell, BMSC, tumor necrosis factor-α, TNF-α, vitamin D, curcumin, resveratrol, enzyme-linked immunosorbent assay, ELISA, inflammation, anti-inflammation, proinflammation, 3D culture system

## Abstract

**Background:**

Chondrocytes are the primary cells responsible for maintaining cartilage integrity and function. Their role in cartilage homeostasis and response to inflammation is crucial for understanding the progression and potential therapeutic interventions for various cartilage-related disorders. Developing an accessible and cost-effective model to generate viable chondrocytes and to assess their response to different bioactive compounds can significantly advance our knowledge of cartilage biology and contribute to the discovery of novel therapeutic approaches.

**Objective:**

We developed a novel, streamlined protocol for generating chondrocytes from bone marrow–derived mesenchymal stem cells (BMSCs) in a 3D culture system that offers significant implications for the study of cartilage biology and the discovery of potential therapeutic interventions for cartilage-related and associated disorders.

**Methods:**

We developed a streamlined protocol for generating chondrocytes from BMSCs in a 3D culture system using an “in-tube” culture approach. This simple pellet-based 3D culture system allows for cell aggregation and spheroid formation, facilitating cell-cell and cell–extracellular matrix interactions that better mimic the in vivo cellular environment compared with 2D monolayer cultures. A proinflammatory chondrocyte model was created by treating the chondrocytes with lipopolysaccharide and was subsequently used to evaluate the anti-inflammatory effects of vitamin D, curcumin, and resveratrol.

**Results:**

The established protocol successfully generated a large quantity of viable chondrocytes, characterized by alcian blue and toluidine blue staining, and demonstrated versatility in assessing the anti-inflammatory effects of various bioactive compounds. The chondrocytes exhibited reduced inflammation, as evidenced by the decreased tumor necrosis factor-α levels, in response to vitamin D, curcumin, and resveratrol treatment.

**Conclusions:**

Our novel protocol offers an accessible and cost-effective approach for generating chondrocytes from BMSCs and for evaluating potential therapeutic leads in the context of inflammatory chondrocyte–related diseases. Although our approach has several advantages, further investigation is required to address its limitations, such as the potential differences between chondrocytes generated using our protocol and those derived from other established methods, and to refine the model for broader applicability and clinical translation.

## Introduction

### Background

The burgeoning field of regenerative medicine has placed a considerable focus on the differentiation of bone marrow–derived mesenchymal stem cells (BMSCs) into chondrocytes [1,2]. This area of research is critical for advancing our understanding of cartilage repair and regeneration and for developing novel therapeutic strategies [3]. Therefore, it is essential to consider the optimal environment for these sophisticated cellular processes. Although cell culture plates have been the standard for differentiation experiments, recent evidence suggests that using tubes for differentiation may better simulate the native chondrocyte habitat and foster more accurate cell-cell and cell–extracellular matrix (ECM) interactions [4]. Consequently, research has shifted its focus toward tube-based, 3D culture systems that more accurately simulate native tissue conditions [5].

Several studies have highlighted the superiority of tube-based culture over conventional 2D cell culture plates. A comparative analysis is presented in Table 1. The rationale behind this innovative approach lies in the unique characteristics of the chondrocyte ECM and the intricacies of cartilage tissue formation. By using tubes for differentiation, we can create a 3D environment that better mimics the native milieu of chondrocytes, fostering improved cell-cell and cell-ECM interactions. This shift in methodology not only enhances our understanding of chondrogenesis but also has the potential to accelerate the development of novel therapeutic strategies for cartilage repair and regeneration.

**Table 1:** Comparison of 2D and 3D cell culture methods.

	Type of cell culture		Key references	Advantage of tube-based cell culture used in this protocol^a^
	2D	3D		
Cell-cell interactions	Limited	Enhanced	Breslin and O'Driscoll [6]	Enhanced cell-cell interactions
Cell–extracellular matrix interactions	Restricted	More accurate	Cukierman et al [7]	Improved mimicry of in vivo conditions
Spatial organization	Flat and monolayer	Complex and multilayered	Edmondson et al [8]	Better representation of tissue architecture
Cell polarization	Mostly absent	Prominent	Théry [9]	Improved cell polarity and functionality
Cell signaling	Altered and oversimplified	More realistic	Bissell and Radisky [10]	More accurate signaling pathways
Cell migration and invasion	Less accurate	Closer to in vivo	Friedl and Alexander [11]	Improved evaluation of cell migration and invasion
Cellular morphology	Flattened and simplified	More in vivo–like	Ravi et al [12]	Enhanced representation of native morphology
Stiffness	High stiffness (approximately 3 × 10^9^ Pa)	Low stiffness (>4000 Pa)	Krausz et al [13]	Low stiffness reduces durotaxis, augmenting cell growth and proliferation and reduction of cellular stress, which may alter gene expression
Gene expression	Altered	More physiologically relevant	Duval et al [14]	Improved gene expression profile
Cellular functions and responses	Limited and oversimplified	More complex and accurate	Edmondson et al [8]	Better simulation of in vivo functionality
Differentiation and stem cell maintenance	Reduced fidelity	Enhanced fidelity	Hockemeyer and Jaenisch [15]	Improved maintenance of cell identity
Cell viability and proliferation	Unreliable representation	More accurate representation	Langhans [16]	Better assessment of cell growth and survival
Drug sensitivity	Less predictive	More predictive	Imamura et al [17]	More accurate drug sensitivity when compared with the 2D system but not as superior when compared with complex 3D techniques such as organoids and scaffold-based systems
Drug exposure	Nonphysiological	Closer to in vivo	Badr-Eldin et al [18]	Improved drug exposure over the 2D system; but when it comes to in vivo mimicry, it may not be as superior as complex 3D techniques such as organoids and scaffold-based systems
Ease of imaging	Enables simple imaging owing to their monolayer structure	Requires sophisticated imaging protocol owing to the complex and multilayered structure	Temple et al [19]	Ease of imaging is high in 2D over 3D cell culture systems, although in-tube–based 3D cell culture systems involving a single type of cell, imaging is relatively easy with high replicability over complex 3D techniques such as organoids and scaffold-based systems
Technical complexity	Low	Higher and varies according to the type of 3D culture system being used	Cacciamali et al [20]	In this protocol, complexity is moderate when compared with a 2D culture system
Scalability	High	Variable and technique dependent	Fang and Eglen [21]	Depends on the 3D cell culture technique but is relatively high for tube-based 3D cell culture techniques. Scalability is especially restricted in scaffold and matrix and microfluidic 3D models
Reproducibility	High	Variable and technique dependent	Duval et al [14]	Typically higher than other 3D cell culture techniques
Cost of experimentation	Low	High—varies with technique	Khalil et al [22]	Typically higher than 2D cell culture techniques, varies with technique but lower when compared with scaffold or matrix, organoid, and microfluidic 3D models
Cost of maintenance	Low	High—varies with technique	Raic et al [23]	Typically higher than 2D cell culture techniques, varies with technique but lower when compared with scaffold or matrix, organoid, and microfluidic 3D models.

^a^The 3D tube-based method for culturing chondrocytes demonstrated in this protocol offers advantages over 2D culture plates, such as enhanced cell-cell interactions, improved extracellular matrix production, and more physiologically relevant cellular responses. Although this tube-based approach is relatively simple compared with other 3D methods such as scaffold-based systems, hydrogels, and organoids, it still possesses many benefits of 3D cell culture systems.

Furthermore, investigating the response of the differentiated chondrocytes to proinflammatory stimuli such as lipopolysaccharide (LPS) is a vital aspect of understanding the intricate mechanisms of cartilage tissue degeneration and inflammation. By creating a proinflammatory model using LPS with the differentiated chondrocytes, we can delve deeper into the cellular and molecular responses that occur during inflammatory conditions such as osteoarthritis. This knowledge is crucial for the development of targeted therapies to mitigate inflammation and promote cartilage regeneration.

### Objective

In this concise research protocol, we outline an uncomplicated approach to differentiate BMSCs into chondrocytes within a 3D culture system. Subsequently, we establish a proinflammatory model using the differentiated chondrocytes for further investigation. This succinct research protocol not only provides a valuable contribution to the field of cartilage tissue engineering but also offers a practical foundation for researchers seeking to explore the potential therapeutic applications and underlying molecular mechanisms in chondrocyte-based proinflammatory models.

## Methods

### Reagents and Equipment

The different reagents and equipment that were used in this study are presented in Textbox 1 in the form of a list for ease of use and reference by researchers.

**Reagents and equipment used in this study.**

**Cell culture**

- Reagents
- Human BMSC line (Addexbio, United States [24])
- Complete MesenCult-ACF Chondrogenic Differentiation Medium (Stem Cell Technologies, Canada [25])
- Phosphate-buffered saline (PBS; Gibco, United Kingdom [26])
  Mesenchymal stem cell growth media (MSCM; Himedia, India [27])
  Fetal bovine serum (Himedia, United States [28])
  Penicillin-streptomycin solution (Himedia, India [29])
  Trypsin and ethylenediaminetetraacetic acid solution (Himedia, India [30])
- Equipment
  - Automated cell counter (DeNovix, Cell Drop, United States [31])
  - Microfuge 20 centrifuge [32]

**Histological section of pellets**

- Reagents
  - Dimethyl sulfoxide [33]
  - Neutral-buffered formalin (TissuePro, United States [34])
- Equipment
  - Microtome (Leica RM2255, Germany [35])

**Staining of chondrocytes**

- Reagents
  - Toluidine blue (C_15_H_16_N_3_S^+^; Abcam, United States [36])
  - Alcian blue (C_56_H_68_C_14_CuN_16_S_4_; Abcam, United States [37])
- Equipment
  - Olympus BX63 microscope (Japan [38])

**Creation and evaluation of proinflammation model**

- Reagents
  - LPS (Thermofisher Scientific, United States [39])
  - Vitamin D (calcitriol; 1,25-dihydroxyvitamin D3 [1,25(OH)2D3]; Abcam, United States [40])
  - Curcumin (MedChem Express, Ukraine [41])
  - Resveratrol (MedChem Express, Ukraine [42])

**Cytokine expression study**

- Reagents
  - Tumor necrosis factor-α (TNF-α) enzyme-linked immunosorbent assay (ELISA) kit (Abcam, United States [43])

- Equipment
  - Microplate reader (Hidex, Finland [44])

### Ethical Considerations

In this study, we conducted experimental procedures exclusively using in vitro methodologies with commercially available cell lines. The absence of animal models or samples, patient samples or data, and the recruitment of human participants ensured minimal risk associated with our research. As such, this study falls under 1 of the exempt review categories defined by the institutional review board regulations of Mohammed Bin Rashid University. For further information and clarification, the institutional review board of the Mohammed Bin Rashid University can be reached at irb@mbru.ac.ae.

As our experiments were conducted using commercially purchased cell lines and did not involve human participants, there was no requirement for informed consent or the inclusion of minors, eliminating the need for parental or guardian consent. In addition, our study did not involve the use of medical records or archived samples. As no human participants were involved, the need for a language waiver was not applicable.

In summary, our study focused on in vitro experiments using commercially available cell lines, negating the involvement of human participants. Consequently, no participant consent was required, and the need for consent, as well as a language waiver which is often required for addressing a form or an agreement while obtaining consent, was waived by the ethics committee. This study complies with human subject research ethics review, exemptions, and approvals, although it primarily involves observations of public behaviors or secondary analyses of research data.

As this study did not involve human participants, the privacy and confidentiality protection descriptions for human participants research, such as statements about data anonymity or deidentification, were not applicable. In addition, because our research did not involve human participants, there was no compensation type or amount provided for human participants research.

**Figure 1:**
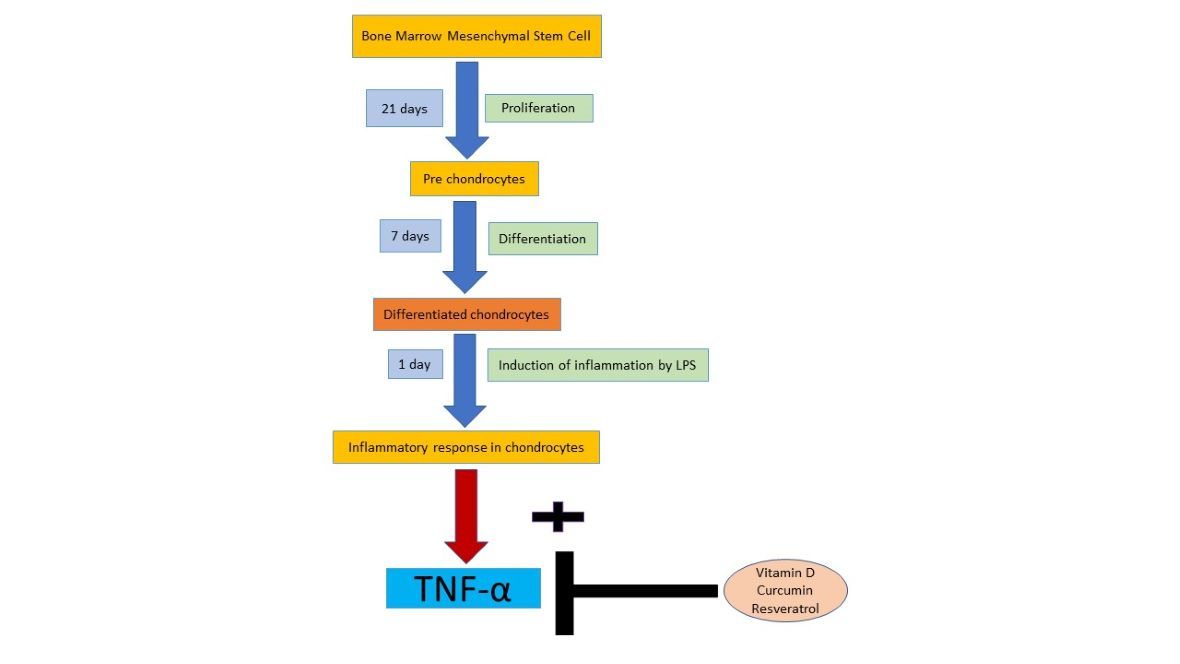
Workflow of this study. LPS: lipopolysaccharide; TNF-α: tumor necrosis factor-α.

The workflow underlying the proof-of-concept study is shown in Figure 1.

### Preparation of Human Bone Marrow Mesenchymal Stem Cell Line for Cell Culture

Upon receiving the human BMSC line in liquid nitrogen, they were immediately washed with PBS to ensure that the cells were free from the cryoprotectant dimethyl sulfoxide. The washed cells were then cultured in MSCM supplemented with 10% fetal bovine serum and 1% penicillin-streptomycin antibiotic solution. After the addition of MSCM, the cells were kept in an incubator at 37 °C and 5% carbon dioxide. The cell culture medium was replaced with fresh medium at an interval of 3 days, and their confluency was appraised. Once the cells reached 90% confluence, the proteolytic enzyme trypsin was added to dislodge the adherent cells from the culture flask. Trypsinization was performed using trypsin and ethylenediaminetetraacetic acid solution. The detached cells were subcultured for further growth in MSCM.

### Cell Culture

In this study, we cultured cells as pellets in 15-mL tubes, instead of using a monolayer culture. Although we did not use a matrix gel or scaffold, our cell culture system can be categorized as a “simple 3D cell culture system.” In this pellet culture system, cells are aggregated and allowed to form spheroids or clusters, promoting cell-cell and cell-ECM interactions that more closely resemble the in vivo cellular environment compared with traditional 2D monolayer cultures [45].

This pellet culture system has several advantages over the 2D cell culture system. First, the 3D cell culture system provides a more physiologically relevant environment for the cells, allowing for better simulation of in vivo conditions, including nutrient diffusion, oxygen gradients, and cell signaling (Table 1) [8]. Second, cell behavior, morphology, and gene expression profiles are often more representative of in vivo conditions in 3D culture systems compared with 2D monolayers (Table 1) [14]. As a result, the pellet culture system may provide more accurate insights into cellular responses to various stimuli, such as drug treatments or the presence of inflammatory molecules, which can be beneficial for therapeutic development and disease modeling.

It is worth noting that although the pellet culture system described in this study represents a simple form of 3D cell culture, more complex 3D culture systems using scaffolds or matrix gels can further enhance the recapitulation of the in vivo microenvironment and may provide additional benefits for various applications [46].

After 5 passages of subculture (passage 5), the BMSCs were used for chondrogenic differentiation. For chondrogenic differentiation, 2 × 10^6^ BMSCs (estimated using an automated cell counter) were pelleted in 15-mL polypropylene tubes after centrifugation at 1000 rpm for 10 minutes. In total, 2 mL of complete MSCM chondrogenic differentiation medium was added to the cell pellet, from which 0.5 mL of the cell suspension was added to each 15-mL polypropylene tube (4 in total) for “in-tube” differentiation. Each of these tubes was centrifuged at 1000 rpm for 10 minutes at 25 °C. Caps of the tubes were gently loosened, and the tubes were incubated at 37 °C in the presence of 5% carbon dioxide to ensure that there were no alterations in the pH of the cell culture media. After 3 days of incubation, 0.5 mL of complete MSCM chondrogenic differentiation medium was added to the tubes, followed by incubation at 37 °C in the presence of 5% CO₂ for 3 days. After incubation, the cell culture medium was carefully aspirated from each tube, and 0.5 mL of fresh MSCM chondrogenic differentiation medium was added. The tubes were further incubated at 37 °C in the presence of 5% CO₂ for 21 days, with intermittent replacement (at 3-day intervals) of cell culture medium with fresh MSCM chondrogenic differentiation medium. After each change of cell culture medium, the differentiating cell pellets were gently “flicked” to ensure that the pellets did not adhere to the tube surfaces. After 21 days of incubation, the cell pellets, which were at the stage of chondrogenic differentiation to prechondrocytes, were further incubated for 7 days.

### Cryopreservation of Differentiated Chondrocytes

Chondrocytes intended for cryopreservation were centrifuged at 1000 rpm for 10 minutes. The cells were then resuspended in the freezing medium and transferred into suitable sized cryovials. The pellets were kept at −80 °C overnight, followed by storage in a liquid nitrogen tank for long-term use.

### Preparation of Histological Section From Cell Pellet

The pellet was transferred to a histology cassette, followed by fixation in 10% neutral-buffered formalin for 24 hours. The cassette along with the pellet were then transferred to 70% ethanol, followed by dehydration in graded ethanol series (25%, 50%, 75%, 90%, 95%, and 100% for 3 minutes each). The clarification steps were carried out in xylene and embedded in paraffin, following a standard embedding protocol. Cell pellets containing chondrocytes were sectioned (6 µm) using a microtome. The sections were mounted on slides. The sections were deparaffinized in 3 changes of xylene, followed by rehydration of the slide sections using a decreasing alcohol series (100%, 95%, 90%, 75%, 50%, and 25% for 3 minutes each), followed by a final rinse with deionized water for 5 minutes.

### Toluidine Blue Staining

Toluidine blue solution was prepared by dissolving 0.1 g of toluidine blue in 100 mL of deionized water. The pH of the solution was adjusted to 4. Following the preparation of slides (as described in detail in the *Preparation of Histological Section From Cell Pellet* section), the slides were stained with toluidine blue and kept for 2 minutes at room temperature. The slides were carefully rinsed with water for 1 minute, followed by dehydration in a graded ethanol series (25%, 50%, 75%, 90%, 95%, and 100% for 3 minutes each). The slides were incubated in xylene for 5 minutes to improve visibility by removal of impurities, and mounted in mounting solution. The slides were then observed using a microscope.

### Alcian Blue Staining

The alcian blue solution was prepared by dissolving 1 g of alcian blue in 3% acetic acid. The pH of the solution was adjusted to 2.5. The preprepared slides (as described in detail in the *Preparation of Histological Section From Cell Pellet* section) containing 6-μm sections of the pellet were placed in 3% acetic acid solution for 3 minutes. The slides were placed in a 1% solution of alcian blue for 30 minutes. The slides were then washed with running water for 1 minute, followed by dehydration in a graded ethanol series (25%, 50%, 75%, 90%, 95%, and 100% for 3 minutes each). The slides were incubated in xylene for 5 minutes to improve visibility by removal of impurities, and mounted in mounting solution. The slides were then observed using a microscope.

### Microscopic Analysis

The Olympus BX63 microscope was used to analyze the staining protocol. The microscope is equipped with Olympus DP80 camera (resolution of 4080 × 3072 pixels and pixel size of 6.45 × 6.45 µm) with Cell Sens Dimension 2.3 software (Olympus Soft Imaging Solutions GmbH). Images were acquired with 20X (numerical aperture 0.45; 1920 × 1200 pixels; 465.079-nm/pixel resolution in both x-axis and y-axis) and 40X (numerical aperture 0.6; 1920 × 1200 pixels; 232.54-nm/pixel resolution in both x-axis and y-axis) objective lenses.

### Creation and Evaluation of the Proinflammation Model

LPS was used to stimulate inflammation in the differentiated chondrocytes to create the proinflammation model. LPS was administered at 10 µg/mL concentration to the differentiated chondrocytes to induce inflammation. The cells were incubated in an incubator at 37 °C and 5% CO₂ concentration for 1 day.

To validate our proinflammation model, we evaluated the anti-inflammatory effects of vitamin D, curcumin, and resveratrol by their dose-dependent inhibition of TNF-α. After 1 day of LPS treatment, LPS-treated chondrocytes were treated with various doses of vitamin D (calcitriol) at 0, 0.01, 0.02, 0.12, and 0.25 µM. Curcumin and resveratrol were used at various concentrations (0, 25, 50, 75, and 100 µM). Following treatment with these nutraceutical leads, the cells were incubated at 37 °C and 5% CO₂ concentration for 1 day.

### Cytokine Expression Study

Expression of TNF-α was assessed in LPS-treated chondrocytes with or without vitamin D, curcumin, and resveratrol. The chondrocyte supernatants were collected by centrifugation at 5000 rpm for 10 minutes. Concentrations of TNF-α were determined through the use of a commercially available ELISA kit. The assay was performed in accordance with the manufacturer’s guidelines. The plates were precoated with human TNF-α. TNF-α levels were measured using a microplate reader at 450 nm, with a reference wavelength of 620 nm. All experiments were performed independently 3 times. All data were reported as mean (SD).

## Results

### Qualitative Evaluation of Differentiated Chondrocytes

**Figure 2:**
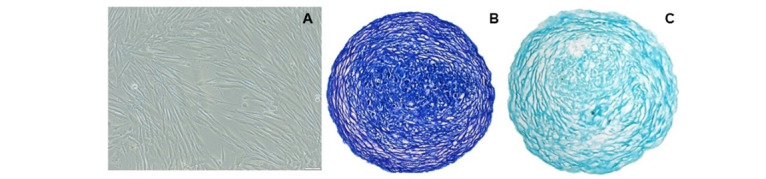
Qualitative analysis of the differentiated chondrocytes. (A) Differentiated chondrocytes obtained from bone marrow–derived mesenchymal stem cells after 28 days; (B) differentiated chondrocytes stained with toluidine blue, and (C) differentiated chondrocytes stained with alcian blue.

Chondrocytes successfully differentiated from human BMSCs (Figure 2A) after culturing in a chondrogenic differentiation medium. The quality of the differentiated chondrocytes was assessed histologically using 2 different dyes: toluidine blue (Figure 2B) and alcian blue (Figure 2C).

Qualitative assessment of successful chondrogenic differentiation of BMSCs was pursued using toluidine blue staining, where the cationic dye specifically binds to the sulfate groups of proteoglycans. A well-differentiated chondrocyte will stain purple owing to the presence of proteoglycans in the ECM, as observed in this study (Figure 2B). The expression of proteoglycan (aggrecan), type II collagen and type X collagen after 21 days of differentiation, was confirmed in the chondrocytes by alcian blue staining (Figure 2C).

### Creation of Proinflammatory Chondrocyte Model and Its Evaluation

Following the generation of high-quality chondrocytes from BMSCs, inflammation was induced in the chondrocytes to generate a “proinflammation model.” Inflammation was induced using LPS, a biomolecule of predominant abundance in the outer membrane of gram-negative bacteria, which is a key pathogenic stimulator for the dysfunctions. During septicemia, circulating LPS, as a pathogen-associated molecular pattern (PAMP) stimulates the innate immune system, leading to local or systemic inflammatory responses. It has also been shown that LPS also stimulates nonimmune cells and initiates the severe inflammatory responses through dedicated receptors. In particular, the innate LPS–pattern recognition receptor, that is, the Toll-like receptor 4 (TLR4), is extensively expressed in cardiomyocytes, which leads to a severe LPS-induced inflammatory response in cardiomyocytes irrespective of the involvement of immune cells [47,48]. TLR4 is also extensively expressed in chondrocytes [49]. Building upon this foundation, we postulated that LPS can trigger proinflammatory reactions in differentiated chondrocytes independent of immune cell participation.

To assess inflammation, the levels of TNF-α in LPS-treated chondrocytes versus LPS-naive chondrocytes (control) were investigated. TNF-α holds particular significance in comparison with other cytokines, with respect to inflammation in chondrocytes. This is primarily because of its well-established role as a key mediator of inflammatory processes in various cell types, including chondrocytes. Several studies have demonstrated the crucial involvement of TNF-α in the pathogenesis of cartilage degradation and osteoarthritis [50-52].

First, TNF-α has been shown to directly promote the production of other proinflammatory cytokines, such as interleukin-1β (IL-1β) and interleukin-6, which subsequently contribute to the inflammatory cascade in chondrocytes [50,53,54]. This amplification of the inflammatory response highlights the central role of TNF-α in this process.

Moreover, TNF-α has been found to induce the expression of matrix metalloproteinases (MMPs) in chondrocytes [55]. MMPs are enzymes responsible for the degradation of the ECM in cartilage tissue, thereby playing a crucial role in cartilage breakdown and the progression of osteoarthritis [56].

In addition, TNF-α can inhibit the synthesis of cartilage matrix components, such as aggrecan and type II collagen, further impairing the structural integrity and function of the cartilage [57,58]. This effect on cartilage homeostasis further underscores the importance of TNF-α as a marker for assessing inflammation in chondrocytes.

In light of these findings, focusing on TNF-α as a marker for evaluating inflammation in chondrocytes is well justified, as it serves as a reliable indicator of the extent of cartilage degradation and the inflammatory status of chondrocytes in various pathological conditions.

Following the addition of LPS, differentiated chondrocytes were incubated for 24 hours, following which the level of TNF-α was assessed by ELISA. LPS-treated chondrocytes expressed 40% more TNF-α, when compared with the LPS-naive chondrocytes (control population of cells treated with PBS instead of LPS).

### Investigating the Effects of Vitamin D, Curcumin, and Resveratrol on Chondrocytes Derived From the “In-Tube” Culture System

After successfully establishing a proinflammatory chondrocyte model, we evaluated its applicability for characterizing potential therapeutic candidates. In this context, we investigated the impact of vitamin D, curcumin, and resveratrol in ameliorating the expression of TNF-α in the proinflammatory chondrocyte model (Table 2).

**Table 2:** Anti-inflammatory response of vitamin D, curcumin, and resveratrol at different doses in the generated proinflammatory chondrocyte model. (Note: Increasing concentrations of the bioceuticals attenuate tumor necrosis factor-α [TNF-α] expression, although vitamin D exhibits the highest potency of inhibition).

Doses (µM)			Percentage of TNF-α expression, mean (SD)		TNF-α expression reduction at max assessed dose (%)	
**Vitamin D**						66.1
	0	100 (0.2)				
	0.01	88 (2.3)				
	0.02	72 (1.7)				
	0.12	58 (1.9)				
	0.25	33.9 (0.9)				
**Curcumin**						61.9
	0	100 (0.8)				
	10	88.4 (2.9)				
	25	72.9 (5.8)				
	50	59.7 (1.2)				
	75	44.3 (6.4)				
	100	38.1 (2.4)				
**Resveratrol**						45.3
	0	100 (1.9)				
	10	92.3 (2.4)				
	25	88.2 (6.8)				
	50	76.2 (3.7)				
	75	63.0 (5.9)				
	100	54.7 (3.0)				

Although the effects of vitamin D on TNF-α are well described in the literature, our study focuses on the unique “in-tube” 3D culture approach for differentiating BMSCs into chondrocytes. This novel method offers significant advantages over traditional 2D culture systems (Table 1), such as improved cell-cell interactions, increased ECM production, and more physiologically relevant cellular responses. Given the unique nature of the “in-tube” culture system, it is essential to verify whether the well-known anti-inflammatory effects of vitamin D on TNF-α are maintained. Using vitamin D in our protocol, we aim to assess the reproducibility and consistency of its effects in an innovative 3D chondrocyte model.

Moreover, the inclusion of vitamin D serves as a positive control to validate the effectiveness of our proinflammatory chondrocyte model, as its effects on TNF-α reduction are well established. By demonstrating that vitamin D effectively reduces TNF-α levels in our “in-tube” culture system, we can confirm the suitability of the model for investigating other potential therapeutics, such as curcumin and resveratrol.

In summary, the use of vitamin D in our protocol is justified by the need to validate the “in-tube” 3D culture system’s ability to replicate well-known anti-inflammatory effects as well as to provide a positive control for evaluating the effectiveness of other nutraceuticals, such as curcumin and resveratrol. The inclusion of vitamin D, along with curcumin and resveratrol, expands our understanding of the potential therapeutic leads for inflammatory chondrocyte–related diseases in the context of our unique 3D culture model.

Vitamin D, specifically its active form, 1,25-dihydroxyvitamin D3 (1,25(OH)2D3), has been shown to exert anti-inflammatory effects in various cell types, including chondrocytes. It modulates the production of proinflammatory cytokines, such as TNF-α and IL-1β, and suppresses the expression of MMPs, which are involved in cartilage degradation [59]. In addition, vitamin D promotes chondrocyte differentiation and enhances the synthesis of ECM components such as type II collagen and aggrecan [60]. Thus, vitamin D can be used to assess the effectiveness of anti-inflammatory agents in a proinflammatory chondrocyte model.

Curcumin, the bioactive component of turmeric, has demonstrated potent anti-inflammatory, antioxidant, and chondroprotective properties [61]. Studies have shown that curcumin can inhibit the expression of proinflammatory cytokines, including TNF-α, IL-1β, and interleukin-6, as well as MMPs in chondrocytes [62-64]. Furthermore, curcumin suppresses the activation of the nuclear factor kappa-light-chain-enhancer of activated B cells (NF-κB), a key transcription factor involved in the regulation of inflammation and cartilage destruction [63]. Therefore, curcumin is an ideal candidate for assessing the efficacy of anti-inflammatory interventions in a proinflammatory chondrocyte model.

Resveratrol, a polyphenol found in grapes, red wine, and other plant sources, exhibits strong anti-inflammatory and antioxidant properties. Research has shown that resveratrol can attenuate inflammation in chondrocytes by inhibiting the production of proinflammatory cytokines and reducing oxidative stress [65]. Moreover, resveratrol suppresses the activation of NF-κB and the expression of MMPs, which are implicated in cartilage degradation [63]. Given its well-documented anti-inflammatory and chondroprotective effects, resveratrol is an appropriate choice for evaluating the performance of anti-inflammatory agents in a proinflammatory chondrocyte model.

In summary, vitamin D, curcumin, and resveratrol possess anti-inflammatory and chondroprotective properties, which make them suitable for assessing the effectiveness of potential therapeutic interventions in a proinflammatory chondrocyte model.

Chondrocytes treated with LPS were exposed to varying concentrations of vitamin D, curcumin, and resveratrol. Each of these nutraceuticals effectively mitigated inflammation, as evidenced by the inverse relationship observed between TNF-α levels and the administered doses of vitamin D, curcumin, and resveratrol (Table 2). This finding has significant implications, as it highlights the potential therapeutic utility of these bioactive compounds in alleviating inflammation and promoting cartilage health in the context of inflammatory chondrocyte conditions. Furthermore, the successful demonstration of the anti-inflammatory effects of vitamin D, curcumin, and resveratrol in this model validates their utility for screening and evaluating other potential therapeutics.

## Discussion

### Principal Findings

In summary, we have described a straightforward and efficient protocol for generating a large quantity of viable chondrocytes derived from BMSCs in a simple pellet-based 3D culture system. This approach, although not using a matrix gel or scaffold, allows for cell aggregation and spheroid formation, facilitating cell-cell and cell-ECM interactions that better mimic the in vivo cellular environment compared with 2D monolayer cultures. However, it is important to note that the pellet culture system is a simple form of 3D cell culture. More complex 3D culture systems using scaffolds or matrix gels can further improve the recapitulation of the in vivo microenvironment, providing additional benefits for various applications.

This approach can also be used to evaluate the effects of potential therapeutic agents on the inflammatory response of chondrocytes. Importantly, this protocol is accessible to all laboratories, including those without readily available patient samples. Furthermore, this “proinflammatory model” can be used to investigate therapeutic targets for diseases involving chondrocytes, such as osteoarthritis.

A notable aspect of this model is its versatility, as it has been effectively used to assess the anti-inflammatory effects of various bioactive compounds such as vitamin D, curcumin, and resveratrol (Table 2). This adaptability allows researchers to explore a wide range of potential therapeutic leads in the context of inflammatory chondrocyte–related diseases, ultimately expanding our understanding of these compounds’ mechanisms of action and their potential for clinical application.

The established model offers several advantages over the existing methods reported in the literature. First, unlike some chondrogenesis models that recommend the use of synthetic or hybrid scaffolds [66], this model does not require scaffold presence. For instance, Sofu et al [67] demonstrated chondrogenesis using a hyaluronic acid scaffold. The incorporation of scaffolds not only adds multiple steps to the experimental process but also increases costs.

Second, our novel chondrogenic differentiation protocol uses an “in-tube” culture system, which reduces the need for cell splitting. Consequently, this minimized the cells’ exposure to degradative enzymes, ensuring the production of higher-quality cells (Figure 2).

Third, by significantly reducing the number of cell-splitting steps, our protocol minimizes human intervention in the chondrogenic differentiation process. This reduction in intervention subsequently decreases the risk of bacterial contamination, genetic alterations, and phenotypic instabilities [68]. Overall, the proposed protocol offers an accessible, cost-effective, and efficient method for generating chondrocytes and for assessing potential therapeutic leads in the context of inflammatory chondrocyte–related diseases.

The chondrocytes derived through this “in-tube” protocol also have diverse applications:

- Tissue engineering and regenerative medicine: The derived chondrocytes can be used for the development of engineered cartilage constructs for cartilage repair and regeneration, particularly in conditions such as osteoarthritis and focal cartilage defects.
- Drug screening and development: The chondrocytes can serve as an in vitro model for evaluating the efficacy and safety of novel therapeutic compounds targeting cartilage-related disorders, potentially accelerating the drug discovery process.
- Disease modeling: The derived chondrocytes can be used to establish in vitro models for investigating the molecular and cellular mechanisms underlying various cartilage-related diseases, providing valuable insights into disease pathogenesis and progression.
- Personalized medicine: By generating patient-specific chondrocytes from autologous BMSCs, our protocol could facilitate the development of personalized treatment strategies tailored to individual patients’ needs, thereby improving therapeutic outcomes and reducing the risk of adverse effects.

### Limitations

Nevertheless, certain aspects of the developed model warrant further investigation. One of the limitations of our proposed protocol is the potential difference between chondrocytes generated using our method and those derived from other established protocols or directly isolated from humans or laboratory animals. Although our simple 3D cell culture system facilitates cell-cell and cell-ECM interactions that more closely resemble the in vivo cellular environment compared with traditional 2D monolayer cultures, it is important to recognize that this system is not as advanced as more complex 3D culture systems using scaffolds or matrix gels. These more sophisticated systems can further improve the recapitulation of the in vivo microenvironment and provide additional benefits for various applications.

Another aspect to consider is the use of single-cell transcriptomics to compare the gene expression profiles of chondrocytes generated using our protocol with those isolated directly from humans or laboratory animals. This analysis would help to validate the methodological strength of our approach and provide valuable insights into the similarities and differences between the generated chondrocytes.

Another limitation of our protocol is that the pellet culture system, although beneficial for cell aggregation and spheroid formation, may not be suitable for all cell types or for studying specific cellular behaviors. Certain cell types may not adhere or proliferate well in this system, and the lack of a defined matrix or scaffold may limit the ability to investigate cell-matrix interactions and cellular migration.

In addition, our protocol has not been extensively tested for scalability, which could be a concern when attempting to generate large quantities of chondrocytes for applications such as tissue engineering or regenerative medicine. Further optimization of the protocol may be required to ensure that the system can be effectively scaled up to meet the demands of these applications.

Moreover, in response to an inflammatory microenvironment, chondrocytes adapt their physiology by modulating the expression of cell surface proteins, collectively referred to as the “surfaceome” [69], and the assortment of transporters and ion channels, known as the “channelome” [70]. It is crucial to determine whether the chondrocytes obtained using this new protocol exhibit surfaceome and channelome profiles consistent with those found in physiological conditions.

In addition, other potential limitations of this study should be acknowledged. For instance, further research is necessary to validate the long-term stability and functionality of the generated chondrocytes in both in vitro and in vivo settings. Moreover, it would be beneficial to explore the effects of other inflammatory mediators on the chondrocytes derived from this protocol to determine their applicability in a broader range of inflammation-driven scenarios. Finally, a comprehensive assessment of the safety and efficacy of the tested bioactive compounds is essential before transitioning to clinical applications. By addressing these limitations, the model used in this study can be refined and expanded to provide a more robust foundation for future research on inflammatory chondrocyte–related diseases.

### Conclusions

In conclusion, the development of our novel, streamlined protocol for generating chondrocytes from BMSCs in a 3D culture system offers significant implications for the study of cartilage biology and the discovery of potential therapeutic interventions for cartilage-related disorders. The “in-tube” culture approach not only allows for the efficient production of viable chondrocytes but also provides a versatile platform for evaluating the anti-inflammatory effects of various bioactive compounds, as demonstrated by the decreased TNF-α levels following treatment with vitamin D, curcumin, and resveratrol. This accessible and cost-effective model holds promise for advancing our understanding of chondrocyte function in cartilage homeostasis and its response to inflammation, which is critical for unraveling the progression of cartilage-related diseases and identifying novel therapeutic strategies. Although our findings contribute to the growing body of knowledge in the field, further investigation is needed to address the limitations of the model used in this study, refine its applicability, and facilitate its translation into clinical practice. By doing so, this innovative approach may ultimately pave the way for the development of more effective and targeted therapies for a range of cartilage-related disorders, ultimately improving patient outcomes and quality of life.

## Abbreviations

BMSC: bone marrow–derived mesenchymal stem cell
ECM: extracellular matrix
ELISA: enzyme-linked immunosorbent assay
IL-1β: interleukin-1β
LPS: lipopolysaccharide
MMP: matrix metalloproteinase
MSCM: mesenchymal stem cell growth media
PAMP: pathogen-associated molecular pattern
PBS: phosphate-buffered saline
TLR4: Toll-like receptor 4
TNF-α: tumor necrosis factor-α
